# Novel 61-bp Indel of *RIN2* Is Associated With Fat and Hatching Weight Traits in Chickens

**DOI:** 10.3389/fgene.2021.672888

**Published:** 2021-07-01

**Authors:** Wujian Lin, Tuanhui Ren, Wangyu Li, Manqing Liu, Danlin He, Shaodong Liang, Wen Luo, Xiquan Zhang

**Affiliations:** ^1^Department of Animal Genetics, Breeding, and Reproduction, College of Animal Science, South China Agricultural University, Guangzhou, China; ^2^Guangdong Provincial Key Lab of Agro-Animal Genomics and Molecular Breeding, Key Laboratory of Chicken Genetics, Breeding and Reproduction, Ministry of Agriculture, Guangzhou, China

**Keywords:** *RIN2*, INDEL, chicken, hatching weight, abdominal fat, transcription factor

## Abstract

The Ras and Rab interactor 2 (*RIN2*) gene, which encodes RAS and Rab interacting protein 2, can interact with GTP-bound Rab5 and participate in early endocytosis. This study found a 61-bp insertion/deletion (indel) in the *RIN2* intron region, and 3 genotypes *II*, *ID*, and *DD* were observed. Genotype analysis of mutation sites was performed on 665 individuals from F_2_ population and 8 chicken breeds. It was found that the indel existed in each breed and that yellow feathered chickens were mainly of the *DD* genotype. Correlation analysis of growth and carcass traits in the F_2_ population of Xinghua and White Recessive Rock chickens showed that the 61-bp indel was significantly correlated with abdominal fat weight, abdominal fat rate, fat width, and hatching weight (*P <* 0.05). *RIN2* mRNA was expressed in all the tested tissues, and its expression in abdominal fat was higher than that in other tissues. In addition, the expression of the *RIN2* mRNA in the abdominal fat of the *DD* genotype was significantly higher than that of the *II* genotype (*P* < 0.05). The transcriptional activity results showed that the luciferase activity of the pGL3-DD vector was significantly higher than that of the pGL3-II vector (*P* < 0.01). Moreover, the results indicate that the polymorphisms in transcription factor binding sites (TFBSs) of 61-bp indel may affect the transcriptional activity of *RIN2*, and thus alter fat traits in chicken. The results of this study showed that the 61-bp indel was closely related to abdominal fat-related and hatching weight traits of chickens, which may have reference value for molecular marker-assisted selection of chickens.

## Introduction

The Ras and Rab interactor 2 (*RIN2*) gene, which encodes RAS and Rab interacting protein 2, functions as a guanine nucleotide exchange factor (GEF). *RIN2* has been shown to interact with Rab5, a small GTPase that participates in early endocytosis (Saito et al., 2002; Grosshans et al., 2006). Rab5 is necessary for the transport of endocytic vesicles to early endosomes (Saito et al., 2002). Deletion of *RIN2* may impair Rab5-related endosome signaling and may also damage the secretion of proteins from the endoplasmic reticulum to the Golgi apparatus or from the Golgi apparatus to the plasma membrane, leading to collagen fiber structure, and phenotypic abnormalities (Syx et al., 2010).

Previous research has shown that *RIN2* syndrome in humans is also called MACS (macrocephaly, alopecia, cutis laxa, and scoliosis) syndrome, a rare hereditary skin disease caused by the loss of the 1-bp homozygote of *RIN2* (Syx et al., 2010; Kameli et al., 2020). A genome-wide selective scan of purebred horses showed that *RIN2* played similar roles in signal transmission, indicating that *RIN2* is under strong artificial selection in racing horses (Moon et al., 2015). Comparative analysis of the genome-wide methylation and transcriptome of the longest muscle in sheep showed that *RIN2* may be a functional gene that affects meat quality traits (Cao et al., 2017). However, no research has reported on the functions of *RIN2* relating to animal production, and its exact functional mechanism in chickens remains unclear.

Indels are a major source of molecular-level variations and have been widely used as molecular markers in the study of economic traits in livestock (Cui et al., 2018). Due to the advantages of convenient detection and remarkable effects, compared with single nucleotide polymorphism (SNP), the indel variants have higher efficiency, and wider application (Li et al., 2017). Indels play an important role in genetic diversity and phenotypic differentiation (Rao et al., 2010; Yan et al., 2014). In poultry, studies have shown that indels are significantly related to chicken growth traits, carcass traits, and other economic traits (Li et al., 2006; Tang et al., 2011; Liang et al., 2019; Liu et al., 2019; Ren et al., 2019). In cattle, indels are significantly related to growth traits and meat quality (Wei et al., 2018; Xu et al., 2018).

In this study, we analyzed whole-genome sequence data, which has been deposited in the archive of the Beijing Institute of Genomics^1^ under accession number CRA000005 (Hou et al., 2020). Chicken *RIN2* is located on chromosome 3 and consists of 20 exons, encoding an 836 amino acid protein. The purpose of this study was to detect internal variation in *RIN2* and clarify the impact of its mutations on the economic traits of chickens.

## Materials and Methods

All animal experiments performed in this study complied with the requirements of the Institutional Animal Protection and Utilization Committee of South China Agricultural University (approval ID: SCAU # 0014). The care and use of animals complied with the local animal welfare laws.

### F_2_ Resource Population

The F_2_ resource population was made up of reciprocal cross between White Recessive Rock (WRR) and Xinghua chickens (XH). WRR are fast-growing broilers, and XH are a slow-growing Chinese native breed. All F_2_ individuals (*n* = 304) were slaughtered at 90 days of age. More information was provided in a previous study (Lei et al., 2005).

### Sample Collection

To confirm the distribution of the *RIN2* genotypic variation in other breeds of chickens, genomic DNA was extracted from a total of 361 healthy individuals from eight breeds. The numbers of samples from each breed were: White Recessive Rock chickens (WRR, *n* = 41), White Leghorn chickens (WHL, *n* = 47), Wenchang chickens (WC, *n* = 48), Qingjiaoma chickens (QJ, *n* = 48), Lushi chickens (LS, *n* = 40), Guangxi yellow chickens (GX, *n* = 46), Gushi chickens (GS, *n* = 44), and Xinghua chickens (XH, *n* = 47).

To detect the expression of *RIN2* mRNA in different tissues, a total of 12 tissues (heart, liver, spleen, lung, kidney, breast muscle, leg muscle, abdomen fat, jejunum, duodenum, hypothalamus, and ovary) were collected from 20-week-old yellow chickens, with four samples of each tissue. DNA was extracted from the blood to determine the genotype. The abdominal fat tissues of 4-week-old yellow chickens from 3 individuals each of the *II*, *ID*, and *DD* genotypes were used to detect the *RIN2* mRNA expression of different genotypes. All tissues were stored at −80°C.

### Genomic DNA Extraction and PCR

Genomic DNA was extracted from blood using a Blood DNA Kit (Omega, Norcross, America). All the primers used in this study were designed using the online tools provided by NCBI^2^ and were synthesized by Beijing TsingKe Company (Supplementary Table 1). PCR was performed in a total volume of 10 μL, including 1 μL of genomic DNA (50 ng/μL), 0.2 μL of each primer (10 μmol/L), 5 μL of 2 × M5 PCR Mix (Yuexing, Guangzhou, China), and 3.6 μL of double distilled H_2_O. The PCR cycle profiles were 95°C for 3 min, 95°C for 25 s, 61°C for 25 s, 72°C for 15 s, and 72°C extension for 5 min for a total of 31 cycles, followed by refrigeration at 4°C. An aliquot (7 μL) of each reaction was electrophoresed on a 2% agarose gel to determine the genotype.

### Diversity Analysis of Different Chicken Breeds

The genotype and allele frequencies of the mutation were calculated directly for different breeds. Hardy-Weinberg equilibrium (HWE) was analyzed using the SHEsis website^3^. The allele number (Ne), genetic indices of heterozygosity (He), polymorphism information content (PIC), and population differentiation were analyzed using PopGene software Version 1.3.1 (Yeh et al., 1999).

### RNA Isolation and cDNA Synthesis

Total RNA from tissues were extracted using TRIzol Reagent (Takara, Dalian, China) following the manufacturer’s protocol. First strand cDNA was synthesized using the Prime Script^TM^ RT Reagent kit (Takara, Dalian, China). Quantitative real-time polymerase chain reaction (qPCR) was used to investigate the expression levels of *RIN2* mRNA in each tissue. qPCR was performed using the CFX96 system (Bio-Rad, Hercules, CA, United States). Three repetitions were performed per sample. The β-*actin* gene was used as an internal control. The primer information is provided in Supplementary Table 1. The qPCR conditions were as follows: 95°C for 5 min, 95°C for 30 s, 60°C for 30 s, and 72°C for 30 s for a total of 35 cycles. The results were analyzed using the 2^−ΔΔ*CT*^method (Schmittgen and Livak, 2008).

### Cell Culture and Cell Transfection

The chicken dermal fibroblast cell line DF-1 was obtained from Guangdong Provincial Key Lab of Agro-Animal Genomics and Molecular Breeding (Guangzhou, China). DF-1 cells were cultured in basic DMEM (Gibco, Carlsbad, CA, United States) with 10% fetal serum (Gibco, Carlsbad, CA, United States) and 1% streptomycin/penicillin (Invitrogen, Carlsbad, CA, United States) at 37°C with 5% CO_2_. A Lipofectamine 3000 kit (Invitrogen, Carlsbad, CA, United States) was used for the transfection of plasmids into cells as described in the next section according to the manufacturer’s protocol. The transfection dose of the plasmids was 0.1 μg/well for 96-well plates.

### Plasmid Construction and Prediction of TFBSs

Based on the sequence diagrams of the chicken *RIN2* 61-bp indel locus (Figure 2), the luciferase reporter vector pGL3 basic (Promega, Madison, WI, United States) was selected, and the pGL3-II vector (161 bp, containing the 61-bp indel) and pGL3-DD vector (100 bp, lacking the 61-bp indel) were constructed by Gene Create (Wuhan, China) to detect the effect of the *RIN2* 61-bp indel.

To detect whether transcription factors bind to the 61-bp indel, the GATA-1 binding sequence (TAAATGCAAA) in the II genotype was deleted based on the prediction results of the TFBSs, and the KO-GATA-1 luciferase reporter vector (151 bp) was constructed by Gene Create. The GCN4 binding sequence (CAAGAGTAAA) was deleted in the II genotype to construct the KO-GCN4 luciferase reporter vector (151 bp). The carrier PRL-TK (Promega, Madison, WI, United States) was used as an internal control in the luciferase reporter system.

Prediction of TFBSs was performed using the Alibaba 2.1^4^ programs.

### Statistical Analysis

Statistical analysis of all the sequence variations and important economic traits related to the F_2_ resource group was performed using SPSS software (version 24.0). The mixed linear models used in the analysis are:

(1) $$\text{Model}:Y_{ijklm}=\mu +G_{i}+S_{j}+H_{k}+f_{l}+e_{ijklm}$$

(2) $$\text{Model}:Y_{ijklm}=\mu +G_{i}+S_{j}+H_{k}+f_{l}+b\left(W_{ijklm}-\bar{W}\right)+e_{ijklm}$$

where, *Y*_*ijklm*_ is the observed value, μ is the overall average, *G*_*i*_ is the fixed effect of the genotype, *f*_*l*_ is the random effect of the family, *S*_*j*_ is the fixed effect of sex, *H*_*k*_ is the fixed effect of the hatch, *b* is the carcass weight regression coefficient, $\bar{W}$ is the average slaughter weight, *W*_*ijklm*_ is the individual slaughter weight, and *e*_*ijklm*_ is the random error term. A *P*-value < 0.05 was considered significant, and Bonferroni’s test was performed to control for multiple comparisons (Ren et al., 2017). Model I was used to assess genotypes related to growth traits and meat quality. Considering the effect of body weight on carcass traits, Model II used carcass weight as a covariate to assess carcass traits.

## Results

### Identification of Genetic Variants in *RIN2*

**FIGURE 1:**
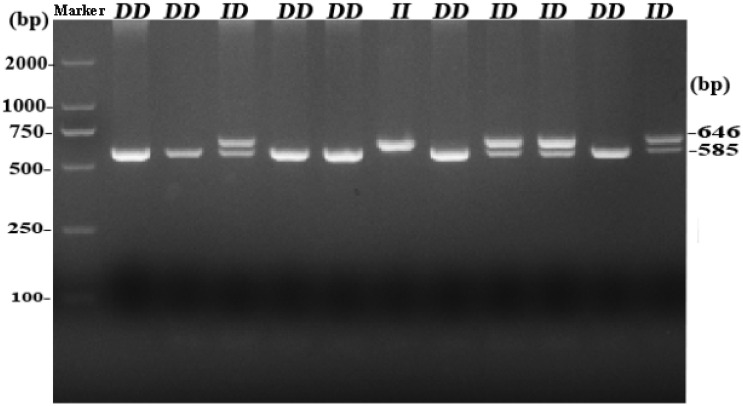
Agarose gel electrophoresis pattern of the *RIN2* 61-bp indel polymorphism.

**FIGURE 2:**
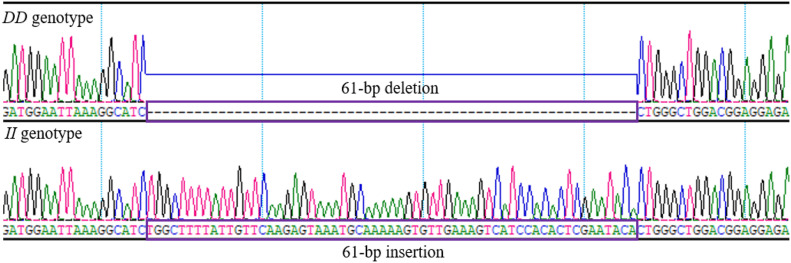
Sequence diagrams of the chicken *RIN2* 61-bp indel locus.

**FIGURE 3:**
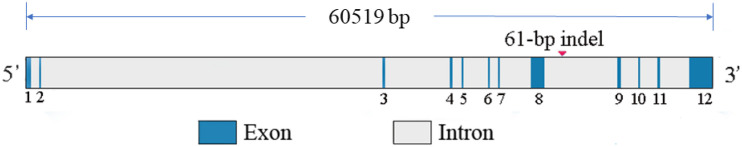
*RIN2* gene structural diagram and the location of the 61-bp indel.

By analyzing whole-genome sequence data, a new 61-bp insertion mutation was found downstream of the intron of *RIN2*. As shown in Figure 1, the indel polymorphism was analyzed by PCR amplification of the region and electrophoresis of the product in a 2.0% agarose gel. Three polymorphisms were identified and named *II* (646 bp), *ID* (646 bp and 585 bp), and *DD* (585 bp). The PCR products were sequenced to pinpoint the location of the insertion (Figure 2). As shown in Figure 3, the 61-bp indel is located in intron 8 of *RIN2*.

### Genetic Parameters of *RIN2* Among F_2_ Resource Populations and Different Breeds

**FIGURE 4:**
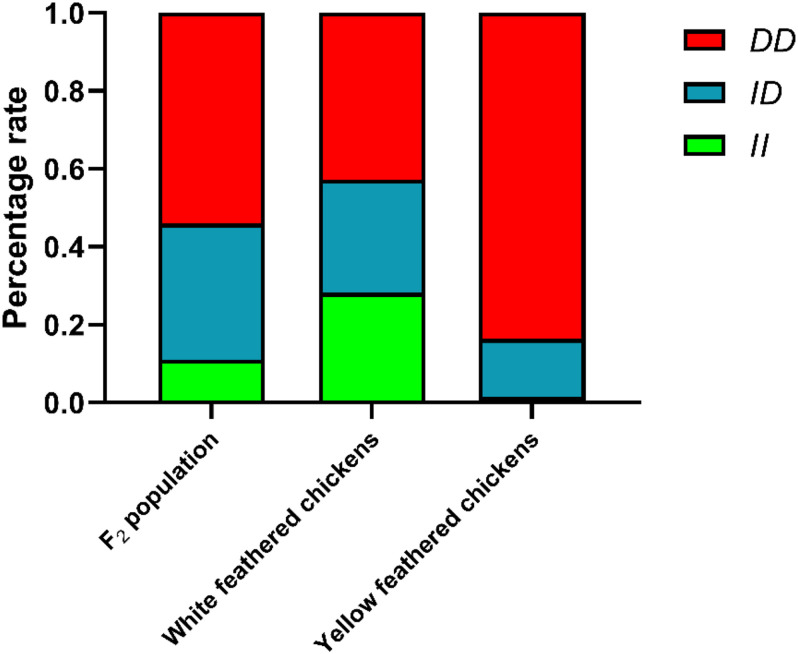
Percentage of different genotypes in different populations. White feathered chickens (White recessive rock chickens and White leghorn chickens), yellow feathered chickens (Wenchang chickens, Qingjiaoma chickens, Lushi chickens, Guangxi yellow chickens, Gushi chickens, and Xinghua chickens).

The genotype and allele frequencies and other genetic parameters associated with the *RIN2* indel locus were calculated for 665 individuals (Table 1). F_2_ population and 8 breeds had the *RIN2* 61-bp insertion mutation, and with the exception of WRR, the allele frequency of *D* was higher than that of *I*. The distribution of genotypes in the different breeds is shown in Figure 4. The frequency of the *II* genotype in white feathered chickens was higher than that in yellow feathered chicken.

**TABLE 1:** Genetic parameters of the 61-bp locus within *RIN2* in chicken breeds.

Breeds	Number	Genotype and gene frequency					He	Ne	PIC	*P-*value
		*II*	*ID*	*DD*	*I*	*D*				
F_2_	304	0.110	0.350	0.540	0.331	0.669	0.406	1.683	0.323	0.014
WRR	41	0.415	0.390	0.195	0.644	0.356	0.476	1.908	0.363	0.249
WHL	47	0.149	0.191	0.660	0.386	0.614	0.370	1.586	0.301	0.001
WC	48	0.000	0.063	0.937	0.032	0.968	0.061	–	0.059	0.823
QJ	48	0.000	0.125	0.875	0.067	0.933	0.117	–	0.110	0.644
LS	40	0.050	0.200	0.750	0.224	0.776	0.255	1.342	0.222	0.173
GX	46	0.000	0.109	0.891	0.058	0.942	0.103	–	0.098	0.697
GS	44	0.000	0.250	0.750	0.144	0.856	0.219	–	0.195	0.343
XH	47	0.042	0.145	0.813	0.115	0.885	0.203	1.255	0.182	0.669

*He, gene heterozygosity; Ne, effective allele numbers; PIC, polymorphism information content; F_2_, F_2_, generation resource population; WRR, White Recessive Rock chickens; WHL, white leghorn chickens; WC, Wenchang chickens; QJ, Qingjiaoma chickens; LS, Lushi chickens; GX, Guangxi yellow chickens; GS, Gushi chickens; XH, Xinghua chickens; *P-*value, *P-*value of the Hardy-Weinberg equilibrium.*

The F_2_, WRR, and WHL breeds showed moderate polymorphism, whereas the remaining breeds showed low polymorphism. The degree of genetic heterozygosity was 0.061–0.476, and the number of effective alleles was 1.255–1.908.

### Differential Selection of the 61-bp Indel Locus

To determine whether differential selection of the *RIN2* 61-bp insertion sequence occurred during the domestication of chickens, the pairwise fixed index (Fst) was used to analyze the breeds differentiation. The analysis showed that the *RIN2* 61-bp site insertion strong genetic differentiation between yellow feathered chickens (WC, QJ, LS, GX, GS, and XH) and WRR (0.2 < Fst < 0.5; Table 2), indicating that the insertion mutation may be selected in WRR. The FST values were lower among the other breeds.

**TABLE 2:** Pairwise fixation index (Fst) of *RIN2* in chicken breeds.

Breeds	WRR	WHL	WC	QL	LS	GX	GS	F_2_
WHL	0.137							
WC	0.398	0.096						
QJ	0.345	0.064	0.005					
LS	0.224	0.014	0.045	0.021				
GX	0.356	0.071	0.003	0.000	0.026			
GS	0.255	0.024	0.031	0.012	0.001	0.015		
F_2_	0.051	0.001	0.04	0.03	0.009	0.032	0.014	
XH	0.270	0.029	0.026	0.008	0.003	0.012	0.000	0.017

*F_2_, F_2_ generation resource population; WRR, white Recessive Rock chicken; WHL, White Leghorn chickens; WC, Wenchang chicken; QJ, Qingjiaoma chicken; LS, Lushi chickens; GX, Guangxi yellow chickens; GS, Gushi chicken; XH, Xinghua chickens.*

### Association of the 61-bp Indel of *RIN2* With Chicken Carcass Traits

In the F_2_ population, the 61-bp indel of *RIN2* was significantly associated with fat traits. Significant correlations were detected with abdominal fat weight, abdominal fat rate and fat width traits (*P* = 0.046, *P* = 0.033, and *P* = 0.005; Table 3). The abdominal fat weight, abdominal fat rate and fat width traits of *DD* genotypes were greater than those of the *ID* and *II* genotypes. There was no significant difference between *ID* and *II* individuals. The 61-bp indel was not significantly associated with other carcass traits (Supplementary Table 2).

**TABLE 3:** Effect of *RIN2* gene polymorphisms on the carcass traits of the F_2_ population.

Traits	Mean ± SE			*P-*value
	*II*	*ID*	*DD*	
FW (mm)	10.262 ± 0.678^a^	11.455 ± 0.439^a^	12.539 ± 0.354^b^	0.005
AFW (g)	21.070 ± 3.893^a.^	24.757 ± 2.876^a^	29.795 ± 2.492^b^	0.046
AFR (%)	1.594 ± 0.279^a^	1.878 ± 0.207^a^	2.235 ± 0.183^b^	0.033

*FW, fat width; AFW, abdominal fat weight; AFR, abdominal fat rate. Different lowercase letters indicate significant differences (*P* < 0.05), and the same letters indicate no difference (*P* > 0.05).*

### Association of the 61-bp Indel of *RIN2* With Chicken Growth and Meat Quality Traits

In the F_2_ population, the 61-bp indel of *RIN2* was significantly related to the hatching weight (*P* = 0.027; Table 4). The hatching weight of chickens of the *II* genotype were greater than those of chickens of the *ID* and *DD* genotypes. There was no significant difference between the *ID* and *DD* genotypes. The other growth traits (shank length, shank diameter, and average daily gain) were not significantly associated with the indel (Supplementary Table 3). The 61-bp indel was not significantly associated with meat quality traits (Supplementary Table 4).

**TABLE 4:** Effect of *RIN2* gene polymorphisms on the growth traits of the F_2_ population.

Traits	Age week	Mean ± SE			*P-*value
		*II*	*ID*	*DD*	
Body weight (g)	0	30.70 ± 0.56^*a*^	29.82 ± 0.48^*b*^	29.79 ± 0.46^*b*^	0.027

*Different lowercase letters indicate significant differences (*P* < 0.05), and the same letters indicate no difference (*P* > 0.05).*

### mRNA Expression Profile of *RIN2* in Chickens

**FIGURE 5:**
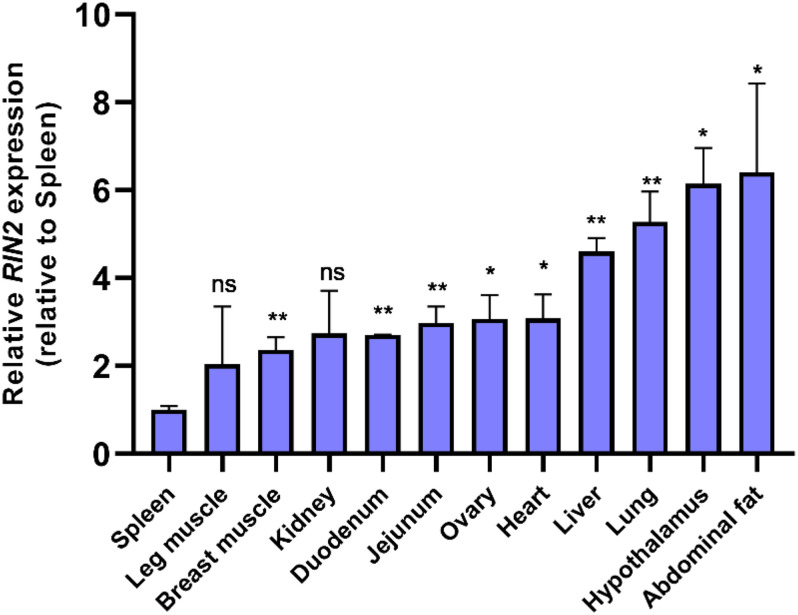
Expression of *RIN2* mRNA in different tissues detected by qPCR (*n* = 4 samples of each tissue). ns, no significant difference (*P* > 0.05); ^∗^, significant difference (*P* < 0.05); **, very significant difference (*P* < 0.01).

The expression of *RIN2* mRNA in various tissues of 20-week-old yellow chickens was studied (Figure 5), and the results showed that *RIN2* was expressed in all the tested tissues. The highest expression was found in abdominal fat and the hypothalamus, followed by the lung and liver, with the lowest expression found in the spleen. The high expression of *RIN2* in abdominal fat suggest that *RIN2* might play a role in the formation of abdominal fat.

### Relative Expression of Different Genotypes of *RIN2*

**FIGURE 6:**
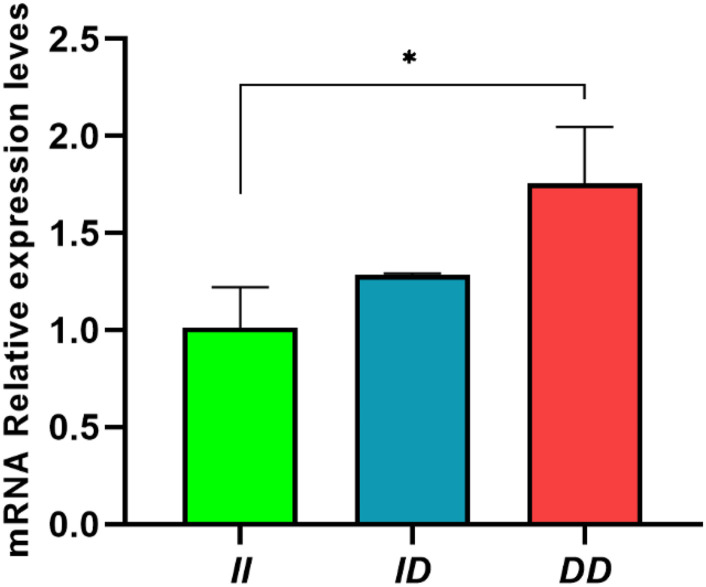
Expression of *RIN2* mRNA in abdomen fat tissue of individuals with different genotypes. Data are presented as the means ± SD (*n* = 3 repetitions for each group). *P < 0.05, significant difference.

In abdominal fat, the *II* genotype showed significantly lower *RIN2* mRNA expression than *DD* genotype (*P <* 0.05; Figure 6). This result indicates that the 61-bp indel may affect the expression of *RIN2* and may affect slaughter traits, such as abdominal fat weight.

### Prediction of TFBSs in the 61-bp Indel

**FIGURE 7:**
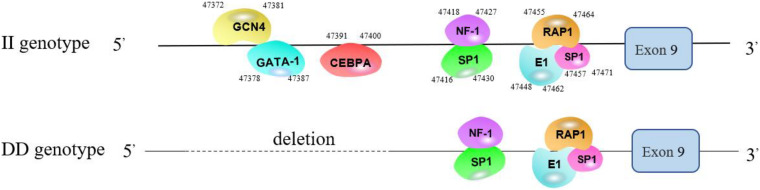
Predicted binding sites of transcription factors in the different genotypes.

According to the prediction results from the Alibaba 2.1 website, the TFBSs of genotype *II* (161 bp) were: GATA-1, the binding sequence of which is TAAATGCAAA at the 47378-47387 site; GCN4, the binding sequence of which is CAAGAGTAAA, at the 47372-47381 site; and CEBPA, the binding sequence of which is TGTTGAAAGT, at the 47391–47400 site. The TFBSs shared with the *DD* genotype (100 bp) were SP1, NF-1, E1, and RAP1 (Figure 7).

### Detection of the Effect of the 61-bp Indel on the Transcriptional Activity of DF-1 Cells

**FIGURE 8:**
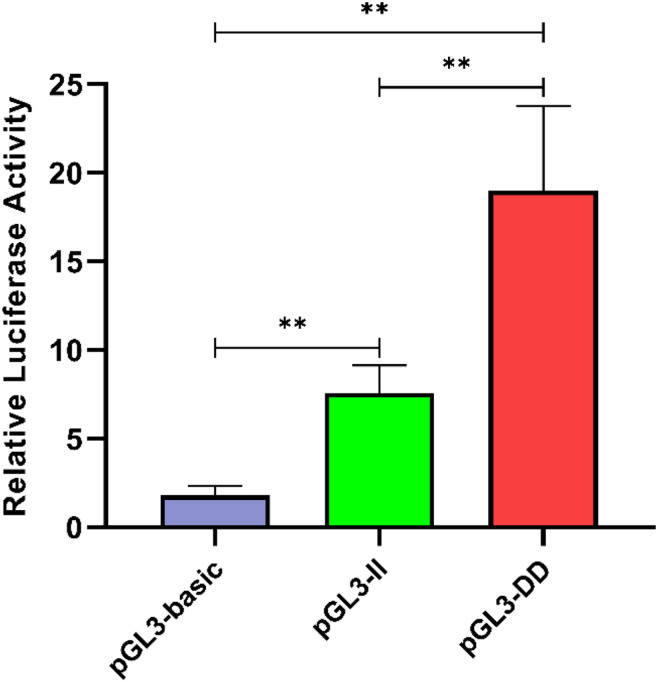
Luciferase activity in DF-1 cells transfected with recombinant plasmids. Data are presented as the means ± SD (*n* = 6 samples of each plasmid). ^∗∗^*P* < 0.01, very significant difference.

To determine the effect of the 61-bp indel on cell transcriptional activity, the pGL3-basic, pGL3-II, and pGL3-DD vectors were cotransfected with PRL-TK into DF-1 cells. The results are shown in Figure 8. The luciferase activity of the pGL3-DD vector was 2.51 times greater than that of the pGL3-II genotype (*P* < 0.01). The luciferase activities of the pGL3-DD, pGL3-II, and pGL3-basic vectors were different (*P* < 0.01). This result suggests that there may be an inhibitor binding site in the *RIN2* 61-bp indel. In fact, the results of the transcription factor site prediction showed that GATA-1 and GCN4 inhibited transcription factor binding (Figure 7).

### Detection of the Effect of Transcription Factors on the Transcription Activity of DF-1 Cells

**FIGURE 9:**
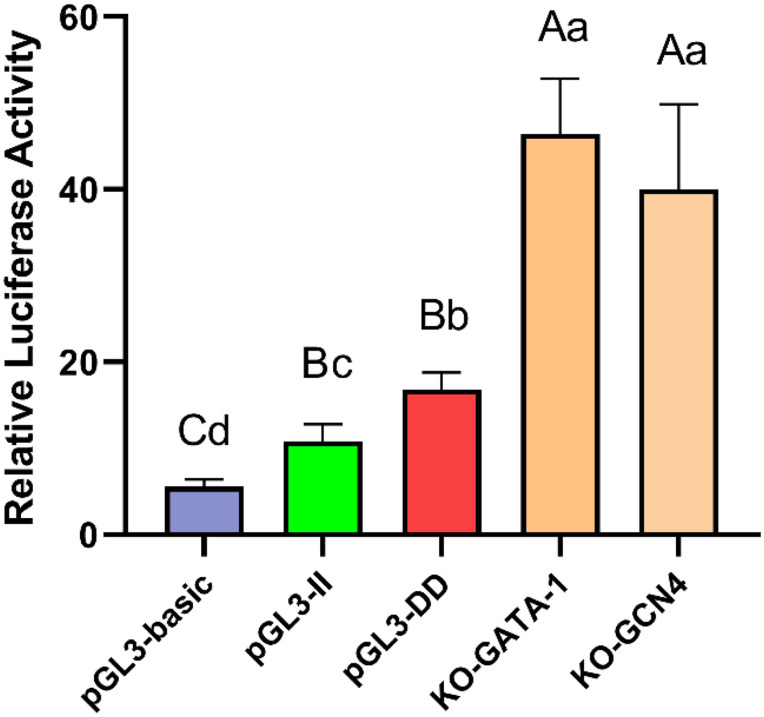
Luciferase activity in DF-1 cells transfected with recombinant plasmids. Data are presented as the means ± SD (*n* = 6 samples of each plasmid). Columns with different letters (A,B) indicate *P* < 0.01, columns with different letters (a,b) indicate *P* < 0.05, and columns with the same letter indicate *P* > 0.05.

To determine whether the GATA-1 or GCN4 transcription factor combined with the 61-bp indel affect cell transcription activity, the GATA-1 and GCN4 binding sequences were deleted and KO-GATA-1 and KO-GCN4 luciferase reporter vectors were constructed. The results are shown in Figure 9. The luciferase activities of the KO-GATA-1 and KO-GCN4 vectors were significantly higher than that of the pGL3-II vector (*P* < 0.01, *P* < 0.01), suggesting that GATA-1 or GCN4 inhibitory transcription factors may combine with the 61-bp indel to reduce cell transcription activity. It is worth noting that the luciferase activities of the KO-GATA-1 and KO-GCN4 vectors were also significantly higher than that of the pGL3-DD vector (*P* < 0.01, *P* < 0.01), suggesting that there may be a 61-bp indel promoting binding sites. In fact, the results of the transcription factor site prediction showed CEBPA TFBSs (Figure 7).

## Discussion

In animal breeding, the discovery of key genes and molecular mechanisms that affect growth traits is an important step in improving breeding efficiency and accelerating the breeding process (Li et al., 2006; Zhang et al., 2014). To enhance the selection effect of the main traits, traditional selection methods can be complemented by gene-assisted selection or molecular marker-assisted selection (MAS). The application of MAS in selection procedures would help in improvement of economic traits in poultry (Han et al., 2010; Sodhi et al., 2013). In this study, a new 61-bp insertion mutation was identified in *RIN2* from the analysis of whole genome resequencing data and PCR product amplification. In the genetic analysis of 665 individuals from F_2_ population and 8 chicken breeds, it was found that there was a 61-bp insertion mutation of *RIN2* in all breeds, and the yellow feathered chickens were mainly of the *DD* genotype (Table 1 and Figure 4). The Fst value showed that the 61-bp insertion genotype had strong genetic differentiation between yellow feathered chickens and WRR (Table 2), indicating that the insertion mutation may have been selected in WRR.

In this study, we found that *RIN2* was expressed in different tissues (Figure 5), which is consistent with previous reports of widespread *RIN2* expression (Wang and Colicelli, 2001). In addition, *RIN2* was highly expressed in abdominal fat and the hypothalamus, suggesting that it may be related to fat deposition and growth. The *RIN2* 61-bp insertion was significantly positive correlated with hatching weight (*P* < 0.05, Table 4). The hatching weight of *II* genotype was greater than that of *ID* and *DD* genotype. During the pregrowth period (1–7 weeks), the weight of genotype *II* was always the highest, while the weight of the *DD* genotype was generally the lowest (Supplementary Table 3).

Hatching weight is the main indicator used to evaluate chick quality (Nitsan et al., 1991). Previous studies have shown that there is a positive correlation between the hatching weight of broilers and their weight at slaughter. For every 1 g increase in hatching weight, the slaughter weight increases by 9–13 g (Singh and Nagra, 2006; Mendes et al., 2011). The economic value of high-hatching weight broilers is also generally higher than that of low-hatching weight broilers (Lara et al., 2005). Analysis of the gene frequency in F_2_ population and 8 chicken breeds revealed that the 61-bp insertion may be highly selected in WRR and WHL (Table 1). We speculate that the 61-bp insertion of *RIN2* may have a positive effect on chicken hatching weight.

It is worth noting that the expression of *RIN2* was highest in abdominal fat tissue. The abdomen is an important area of fat deposition in chickens. Abdominal fat weight is highly related to total body fat deposition in chickens and can be used as an index for selecting chicken fat deposition. Previous studies have found that excessive fat deposits in modern commercial broiler breeds can waste much feed while reducing slaughter rates and economic benefits (Ling et al., 2015; Zhang et al., 2017). For consumers, eating broilers that accumulate too much fat may cause human obesity or other diseases (Khatun et al., 2017). Therefore, the excessive deposition of abdominal fat in chickens has become one of the problems that needs to be solved in broiler production.

The correlation analysis results indicated that the abdominal fat weight and abdominal fat rate of *II* genotype individuals were significantly lower than those of *ID* and *DD* genotype individuals (*P* < 0.05, Table 3). Quantitative analysis of *RIN2* mRNA expression in abdominal fat tissue from individuals of different genotypes showed that the expression in the *II* genotype individuals was significantly lower than *DD* genotype individuals (*P* < 0.05; Figure 6). The transcription activity results showed that the luciferase activity of the pGL3-DD vector was 2.51 times greater than that of the pGL3-II vector (*P* < 0.01, Figure 8). This finding suggests that the 61-bp indel may work in combination with inhibitory transcription factors, resulting in a decrease in the transcriptional activity of *RIN2*, and decreases in abdominal fat weight and abdominal fat rate.

Introns not only enhance genes but also inhibit the expression of some genes (Ding et al., 2006). Studies have shown that a nucleotide mutation in intron 3 of the *IGF2* gene affects muscle growth in pigs, resulting in a decrease in fat content (Van Laere et al., 2003). An inverted repeat sequence in intron 1 of the *Col1A1* gene can hinder gene transcription (Hormuzdi et al., 1998). The newly discovered 10-bp indel in the non-coding region of the *PAX7* gene significantly reduces the expression of *PAX7*, thereby reducing the early growth weight Chinese cattle (Xu et al., 2018).

The transcription factors predicted to bind to the 61-bp indel included the GATA-1 and GCN4 transcription factors (Figure 7). GATA-1 can significantly inhibit the activity of the Nanog promoter (Li et al., 2015). GATA-1 mediates the inhibition of PU.1 gene transcription in human acute myeloid leukemia erythroleukemias (burda et al., 2016). When GCN4 is overexpressed in yeast, the gene-encoding ribosomal protein is inhibited (Mittal et al., 2017). GATA-1 and GCN4 may be inhibitory transcription factors that inhibit the transcriptional activity of target genes.

The GATA-1 and GCN4 TFBSs were deleted, and the constructed luciferase reporter vector was tested; it was found that the luciferase activities of the KO-GATA-1 and KO-GCN4 vectors were significantly higher than that of the pGL3-II vector (*P* < 0.01, *P* < 0.01, Figure 9), suggesting the GATA-1 and GCN4 inhibitory transcription factors may bind to the 61-bp indel, reducing the transcription activity of *RIN2*. To determine how inhibitory transcription factors affect the transcriptional activity of *RIN2*, further research is needed. It is also worth noting that, the transcription factor predicted to bind to the 61-bp indel was the CEBPA transcription factor (Figure 7), and the luciferase activities of the KO-GATA-1 and KO-GCN4 vectors were significantly higher than that of the pGL3-DD vector (*P* < 0.01, *P* < 0.01, Figure 9). KO-GATA-1 and KO-GCN4 lack the binding sites for GATA-1 or GCN4 but not for CEBPA, but the pGL3-DD vector lacks the CEBPA binding site. It is speculated that the 61-bp indel might also include a binding site that promotes transcription factors. However, the effect of promoting transcription factor binding on the transcriptional activity of *RIN2* requires further research.

Based on the above results, it is speculated that the *RIN2* 61-bp insertion might have a negative effect on chicken fat traits. The 61-bp indel may work in combination with the inhibitory transcription factors GATA-1 and GCN4 to reduce the transcriptional activity of *RIN2*, resulting in a decrease in abdominal fat weight and abdominal fat rate. However, the specific mechanisms require further testing.

## Conclusion

The results of this study indicate that the 61-bp indel of *RIN2* is associated with hatching weight and abdominal fatness traits in F_2_ reciprocal cross chickens. *RIN2* mRNA is expressed in all tissues, but is most highly expressed in abdominal fat. The *RIN2* 61-bp insertion may have a negative effect on chicken fat traits and a positive effect on chicken hatching weight; indels may be potential molecular markers in poultry breeding and QTL identification studies.

## Data Availability Statement

The original contributions presented in the study are publicly available. This data can be found here: whole-genome sequence data has been deposited in the archive of Beijing Institute of Genomics (https://bigd.big.ac.cn/gsa/) under the accession number CRA000005.

## Ethics Statement

The animal study was reviewed and approved by the Institutional Animal Protection and Utilization Committee of South China Agricultural University.

## Author Contributions

WJL, TR, and XZ: conceptualization. WJL, TR, WYL, and ML: data curation. WJL and TR: formal analysis and software. XZ: funding acquisition and project administration. WL and SL: investigation. TR, WYL, and ML: methodology. DH, WL, and XZ: resources. DH and SL: supervision. WJL: writing—original draft. WJL and XZ: writing—review and editing. All authors have read and agreed to the published version of the manuscript.

## Conflict of Interest

The authors declare that the research was conducted in the absence of any commercial or financial relationships that could be construed as a potential conflict of interest.

## Footnotes

1 https://bigd.big.ac.cn/gsa/

2 http://www.ncbi.nlm.nih.gov/tools/primer-blast/

3 http://analysis.bio-x.cn/myAnalysis.php

4 http://www.gene-regulation.com/pub/programs/alibaba2/index.html

## Supplementary Material

Supplementary Table 1 Details of the primer pairs for the chicken *RIN2* gene.

Supplementary Table 2 Effect of *RIN2* gene polymorphisms on the carcass traits of the reciprocal cross F_2_ population.

Supplementary Table 3 Effect of *RIN2* polymorphisms on the growth traits of the reciprocal cross F_2_ population.

Supplementary Table 4 Effect of *RIN2* polymorphisms on the meat quality of the reciprocal cross F_2_ population.
