# Supplementary material for: Novel 61-bp Indel of RIN2 Is Associated With Fat and Hatching Weight Traits in Chickens
Source: Front Genet. 2021 Jul 1;12:672888. doi: 10.3389/fgene.2021.672888 (PMC8280519; doi:10.3389/fgene.2021.672888)
Supplement: Supplementary Table 1 — Details of the primer pairs for the chicken RIN2 gene. [file Table_1.DOCX]

Supplementary Material

| **Table S1.** Details of the primer pairs for the chicken *RIN2* gene. | | | |  |
| --- | --- | --- | --- | --- |
| **Gene** | **Primer (5´-3´)** | **Tm (°C)** | **Size (bp)** | |
| *RIN2* | F: AGTCTAGCTCTGGTCGTGTG R: CACCGCACCTGATATCCCAC | 61 | 585/646 | |
| *RT-RIN2* | F: GCAGATTTGCGAAGTCTAGTAAAGA R: AATATCCCAGGAGGCTGTGC | 60 | 212 | |
| *β-actin* | F: GACTGACCGCGTTACTCCCA R: CCAACCATCACACCCTGATGTC | 60 | 166 | |

Note: *RIN2* primers are used for PCR amplification to analyze indel polymorphisms and the percentage of different genotypes in different varieties, *RT-RIN2* primers are used to analyze the *RIN2* mRNA expression level of different genotypes, and *β-actin* primers are used as internal reference controls for qPCR.

| **Table S2.** Effect of *RIN2* gene polymorphisms on the carcass traits of the reciprocal cross F_2_ population. | | | | |
| --- | --- | --- | --- | --- |
|  |  |  |  |  |
| **Traits** | **Mean ± SE** | | | ***P-*value** |
|  | *II* | *ID* | *DD* |  |
| LWBS(kg) | 1.459 ± 0.048 | 1.513 ± 0.032 | 1.507 ± .027 | 0.477 |
| SL(mm) | 86.168 ± 1.395 | 87.588 ± 1.000 | 86.585 ± 0.851 | 0.335 |
| HW(mm) | 30.459 ± 0.303 | 30.539 ± 0.189 | 30.223 ± 0.149 | 0.341 |
| BW(mm) | 67.802 ± 1.281 | 67.236 ± 0.812 | 66.032 ± 0.646 | 0.303 |
| BD(mm) | 91.346 ± 1.805 | 94.422 ± 1.251 | 95.150 ± 1.042 | 0.106 |
| BL(cm) | 22.378 ± 0.306 | 22.853 ± 0.203 | 22.830 ± 0.165 | 0.253 |
| BAW(°) | 62.297 ± 1.099 | 60.677 ± 0.770 | 60.592 ± 0.646 | 0.231 |
| CW(g) | 1287.932 ± 45.627 | 1331.022 ± 30.010 | 1349.877 ± 24.290 | 0.437 |
| SEW(g) | 1187.427 ± 40.999 | 1224.958 ± 27.534 | 1224.559 ± 22.533 | 0.607 |
| EW(g) | 1039.602 ± 36.418 | 1060.787 ± 24.599 | 1057.952 ± 20.195 | 0.821 |
| BMW(g) | 90.279 ± 3.592 | 91.980 ± 2.498 | 90.133 ± 2.086 | 0.695 |
| LMW(g) | 113.353 ± 4.483 | 115.817 ± 3.071 | 116.010 ± 2.541 | 0.820 |
| WW(g) | 66.207 ± 2.094 | 66.214 ± 1.375 | 63.747 ± 1.113 | 0.227 |
| HNW(g) | 120.951 ± 5.376 | 122.392 ± 3.695 | 124.838 ± 3.066 | 0.730 |
| SCW(g) | 29.175 ± 1.348 | 30.236 ± 0.951 | 28.819 ± 0.801 | 0.244 |
| HLGW(g) | 65.821 ± 2.344 | 68.483 ± 1.610 | 68.835 ± 1.341 | 0.408 |
| SIL (cm) | 137.881 ± 3.795 | 140.549 ± 2.932 | 138.340 ± 2.618 | 0.465 |

Note: liveweight before slaughtering (LWBS), shank length (SL), head width (HW), breast width (BW), breast depth (BD), body length (BL), breast angle width (BAW), carcass weight (CW), semi-evisceration weight (SEW), evisceration weight (EW), breast muscle weight (BMW), leg muscle weight (LMW), wing weight (WW), head and neck weight (HNW), shank and claws weight (SCW), weights of heart, liver, and gizzard (HLGW), small intestine length (SIL).

| **Table S3.** Effect of *RIN2* polymorphisms on the growth traits of the reciprocal cross F_2_ population. | | | | | |
| --- | --- | --- | --- | --- | --- |
|  |  |  |  |  |  |
| **Traits** | **Age week** | **Mean ± SE** | | | ***P-*value** |
|  |  | *II* | *ID* | *DD* |  |
| Body weight | 0 | 30.70 ± 0.56a | 29.82 ± 0.48b | 29.79 ± 0.46b | 0.027 |
| (g) | 1 | 60.52 ± 1.73 | 58.77 ± 1.29 | 59.50 ± 1.13 | 0.403 |
|  | 2 | 125.12 ± 3.65 | 123.66 ± 2.70 | 122.84 ± 2.34 | 0.810 |
|  | 3 | 213.71 ± 6.57 | 209.35 ± 4.81 | 208.37 ± 4.11 | 0.685 |
|  | 4 | 311.02 ± 10.29 | 308.69 ± 7.52 | 307.17 ± 6.41 | 0.930 |
|  | 5 | 455.47 ± 15.05 | 433.91 ± 10.41 | 430.24 ± 8.82 | 0.218 |
|  | 6 | 577.27 ± 20.13 | 566.25 ± 14.26 | 566.41 ± 12.12 | 0.817 |
|  | 7 | 718.14 ± 23.78 | 703.95 ± 16.68 | 696.24 ± 13.96 | 0.661 |
|  | 8 | 854.94 ± 28.23 | 852.97 ± 19.65 | 844.54 ± 16.27 | 0.897 |
|  | 9 | 1009.29 ± 39.13 | 1002.50 ± 24.27 | 1007.47 ± 20.81 | 0.969 |
|  | 10 | 1089.07 ± 39.60 | 1121.53 ± 27.74 | 1106.69 ± 21.80 | 0.666 |
|  | 11 | 1297.25 ± 45.42 | 1316.65 ± 31.59 | 1305.99 ± 25.20 | 0.881 |
|  | 12 | 1421.31 ± 65.04 | 1454.58 ± 39.40 | 1464.44 ± 31.59 | 0.819 |
| Shank length | 6 | 60.83 ± 0.90 | 61.06 ± 0.61 | 59.95 ± 0.51 | 0.203 |
| (mm) | 7 | 67.86 ± 1.30 | 68.28 ± 0.75 | 67.03 ± 0.64 | 0.320 |
|  | 8 | 71.80 ± 0.96 | 72.62 ± 0.66 | 71.62 ± 0.53 | 0.266 |
|  | 9 | 72.68 ± 4.47 | 74.95 ± 3.90 | 74.22 ± 3.77 | 0.600 |
|  | 10 | 81.76 ± 1.23 | 81.96 ± 0.88 | 81.11 ± 0.71 | 0.609 |
|  | 11 | 89.73 ± 1.85 | 89.68 ± 0.94 | 87.32 ± 0.76 | 0.139 |
|  | 12 | 88.18 ± 1.53 | 88.550 ± 0.94 | 87.83 ± 0.76 | 0.761 |
| Shank diameter | 6 | 7.84 ± 0.16 | 7.92 ± 0.12 | 7.81 ± 0.10 | 0.499 |
| (mm) | 7 | 8.58 ± .21 | 8.53 ± 0.12 | 8.48 ± 0.10 | 0.902 |
|  | 8 | 8.80 ± 0.19 | 8.80 ± 0.14 | 8.68 ± 0.12 | 0.624 |
|  | 9 | 9.12 ± 0.34 | 9.19 ± 0.18 | 9.16 ± 0.15 | 0.968 |
|  | 10 | 9.299 ± 0.211 | 9.503 ± 0.155 | 9.348 ± 0.128 | 0.391 |
|  | 11 | 9.52 ± 0.36 | 9.97 ± 0.20 | 9.78 ± 0.16 | 0.375 |
|  | 12 | 10.12 ± 0.27 | 10.07 ± 0.20 | 9.92 ± 0.17 | 0.604 |
| Average daily | 0-4 | 10.03 ± .37 | 9.98 ± 0.27 | 9.90 ± 0.23 | 0.925 |
| gain (g/week) | 4-8 | 19.40 ± 0.77 | 19.42 ± 0.52 | 19.22 ± 0.42 | 0.933 |

Note: Different lowercase letters of the means superscript show significant differences (*P* < 0.05), the same letters show no difference (*P* > 0.05).

| **Table S4.** Effect of *RIN2* polymorphisms on the meat quality of the reciprocal cross F_2_ population. | | | | |
| --- | --- | --- | --- | --- |
|  |  |  |  |  |
| **Traits** | **Mean ± SE** | | | ***P-*value** |
|  | *II* | *ID* | *DD* |  |
| LML | 61.69 ± 1.67 | 64.97 ± 1.07 | 64.36 ± 0.85 | 0.156 |
| BML | 54.58 ± 1.72 | 56.82 ± 1.03 | 54.93 ± 0.81 | 0.225 |
| LMS(N) | 69.15 ± 4.62 | 76.92 ± 2.62 | 71.49 ± 2.09 | 0.178 |
| BMS(N) | 34.19 ± 2.05 | 32.89 ± 1.29 | 31.62 ± 1.03 | 0.464 |
| LMPH | 6.05 ± 0.08 | 6.03 ± 0.06 | 6.11 ± 0.04 | 0.459 |
| BMPH | 6.13 ± 0.11 | 6.12 ± 0.07 | 6.09 ± 0.05 | 0.897 |
| LME(mS/cm) | 1.88 ± 0.17 | 1.95 ± 0.10 | 2.10 ± 0.08 | 0.343 |
| BME(mS/cm) | 6.90 ± 0.70 | 5.91 ± 0.46 | 6.06 ± 0.37 | 0.355 |
| WL(%) | 18.22 ± 2.81 | 16.04 ± 1.88 | 19.76 ± 1.51 | 0.309 |
| BMDM(%) | 26.19 ± 0.18 | 26.20 ± 0.11 | 26.20 ± 0.08 | 0.998 |
| LMDM(%) | 25.15 ± 0.28 | 25.45 ± 0.19 | 25.79 ± 0.14 | 0.084 |
| BMCP(%) | 25.08 ± 0.20 | 24.95 ± 0.12 | 24.86 ± 0.09 | 0.553 |
| LMCP(%) | 20.69 ± 0.17 | 20.60 ± 0.11 | 20.76 ± 0.08 | 0.472 |
| BMCF(%) | 0.81 ± 0.10 | 1.02 ± 0.07 | 1.08 ± 0.05 | 0.069 |
| LMCF(%) | 4.06 ± 0.28 | 4.30 ± 0.20 | 4.45 ± 0.16 | 0.381 |

Note: leg muscle color L value (LMC),breast muscle color L value (BML), leg muscle shear force (LMS), breast muscle shear force (BMS), leg muscle PH value (LMPH), breast muscle PH value (BMPH), leg muscle electrical conductivity (LME), breast muscle electrical conductivity (BME), water loss rate (WL), breast muscle dry matter content (BMDM), leg muscle dry matter content (LMDM), breast muscle crude protein content (BMCP), leg muscle crude protein content (LMCP), breast muscle crude fat content (BMCF), leg muscle crude fat content (LMCF).
